# Neurotensin Decreases the Proinflammatory Status of Human Skin Fibroblasts and Increases Epidermal Growth Factor Expression

**DOI:** 10.1155/2014/248240

**Published:** 2014-08-11

**Authors:** Lucília Pereira da Silva, Bruno Miguel Neves, Liane Moura, Maria Teresa Cruz, Eugénia Carvalho

**Affiliations:** ^1^Faculdade de Ciências e Tecnologia, Universidade de Coimbra, 3000-456 Coimbra, Portugal; ^2^Centro de Neurociências e Biologia Celular, Universidade de Coimbra, 3004-504 Coimbra, Portugal; ^3^Faculdade de Farmácia, Universidade de Coimbra, 3000-548 Coimbra, Portugal; ^4^Associação Portuguesa de Diabetes (APDP), 1250-189 Lisboa, Portugal

## Abstract

Fibroblasts colonization into injured areas during wound healing (WH) is responsible for skin remodelling and is also involved in the modulation of inflammation, as fibroblasts are immunologically active. Herein, we aimed to determine neurotensin effect on the immunomodulatory profile of fibroblasts, both in homeostatic and inflammatory conditions. Neurotensin mediated responses occurred through NTR1 or NTR3 receptors, while under inflammatory conditions NTR1 expression increase seemed to modulate neurotensin responses. Among different immunomodulatory genes, CCL11, IL-8, and IL-6 were the most expressed genes, while CCL4 and EGF were the less expressed genes. After neurotensin exposure, IL-8 mRNA expression was increased while CCL11 was decreased, suggesting a proinflammatory upregulation and chemoattractant ability downregulation of fibroblasts. Under inflammatory conditions, gene expression was significantly increased. After neurotensin exposure, CCL4 and IL-6 mRNA expression were decreased while CCL11 was increased, suggesting again a decrease in the chemoattractant capacity of fibroblasts and in their proinflammatory status. Furthermore, the expression of EGF, a crucial growth factor for skin cells proliferation and WH, was increased in all conditions. Overall, neurotensin, released by nerve fibers or skin cells, may be involved in the decrease of the chemotaxis and the proinflammatory status in the proliferation and remodelling phases of WH.

## 1. Introduction

Neuropeptides can be produced by skin cells or be released by peripheral nerves into the skin, where they bind to respective receptors stimulating different signalling pathways and cellular responses [1, 2]. Within the cellular responses and events that can be modulated in the skin by neuropeptides, inflammatory processes are some of the most important. However, the role of neurotensin (NT) in the modulation of skin inflammation is still unclear.

The first report demonstrating NT-positive fibbers in the skin was in 1983 [3] and it was later confirmed by Donelan et al. in 2006 [4]. The interest in NT first appeared when its influence on the pathogenesis of skin disorders exacerbated by stress was discovered [5]. In fact, it has been observed that in many skin disorders worsened by stress, the number and activation status of mast cells increase. Accordingly, NT has been shown to increase the number and activation of mast cells in a skin pathogenesis, such as psoriasis [6], which is in agreement with several other reports showing that mast cell degranulation is triggered by stress and via neurotensin [7–9]. Moreover, skin vascular permeability induced by corticotropin-releasing hormone (CRH) on mast cells has been shown to occur through a neurotensin-dependent mechanism [4]. In fact, human mast cells are able to synthesize a neurotensin precursor, secrete bioactive NT-like peptide(s), and express NT receptor NTS1 [10]. Besides mast cells, neurotensin has also been shown to enhance the chemotaxis capacity of lymphocytes and to limit the growth of cutaneous T-cell lymphoma tumor cells [11]. Most recently, we have demonstrated that NT can modulate inflammatory events on a skin dendritic cell line [12]. Fetal-skin dendritic cells expressed both NTR1 and NTR3 and neurotensin was able to downregulate the activation of inflammatory signalling pathways and the expression of cytokines IL-6, TNF-*α*, and IL-10, as well as vascular endothelial growth factor (VEGF), while upregulating the survival pathway ERK and epidermal growth factor (EGF) expression [12].

In spite of these results not much has been studied regarding NT effects in fibroblasts. Therefore, in the present study we used a cell line established from the skin of normal newborn human foreskin fibroblasts. Fibroblasts are a heterogeneous population of cells of mesenchymal origin which support the development, repair, and homeostasis of their resident tissue. Dermal fibroblasts play a key role in extracellular matrix (ECM) deposition, epithelial-mesenchymal interactions, and wound healing [13]. They also contribute to the immune regulation of the skin, producing and releasing chemokines IL-8 and CCL5, cyclooxygenases, and prostanoids, after activation by inflammatory agents, such as bacterial agents [14]. By using this cell line, we intended to decrease the variability of the donors, although lacking the variability of skin from different body locations. Using fibroblasts from the foreskin ensures the use of cells from a skin area replenished of a rich and complex network of nerves with neurophysiologic parameters [15, 16].

The main aim of this work was to unravel the effect of neurotensin and its chemoattractant capacity in newborn foreskin fibroblasts, in both homeostatic and inflammatory conditions, in order to understand the possible physiological role of this neuropeptide in skin wound healing as well as speculate about the role of this neuropeptide in other skin pathologies.

## 2. Materials and Methods

### 2.1. Materials

Lipopolysaccharide (LPS) from* Escherichia coli* (serotype 026:B6) was obtained from Sigma Chemical Co. (St. Louis, MO, USA), NT was obtained from Bachem (Weil am Rhein, Germany), 30% Acrylamide/BisSolution 29 : 1, TEMED, and SYBR Green were obtained from Bio-Rad, and High Capacity cDNA Reverse Transcription kit was obtained from Applied Biosystems.

The protease inhibitor cocktail (Complete Mini) and the phosphatase inhibitor cocktail (PhosSTOP) were obtained from Roche (Carnaxide, Portugal). Bicinchoninic acid (BCA) kit assay was obtained from Novagen. The polyvinylidene difluoride (PVDF) membranes and the antibody against *β*-actin were purchased from Millipore Corporation (Bedford, MA, USA). The polyclonal antibodies against NTR1(H-130), NTR2(H-19), and NTR3(H-300) were purchased from Santa Cruz (Frilabo), references sc-15311, sc-31696, and sc-30217, respectively. The alkaline phosphatase-linked secondary antibody and the enhanced chemifluorescence (ECF) reagent were obtained from GE Healthcare (Carnaxide, Portugal). The Vectashield mounting medium was purchased from Vector Inc. (Burlingame, CA, USA), the Alexa Fluor 488 antibody was purchased from Molecular Probes (Eugene, OR, USA), and the Alexa Fluor 555 phalloidin antibody was purchased from Invitrogen (Barcelona, Spain). TRIzol was obtained from Invitrogen; diethyl pyrocarbonate (DEPC) was acquired from AppliChem. Methanol, ethanol, and isopropanol were obtained from Merck. All primers were obtained from MWG Biotech (Ebersberg, Germany). All other reagents were purchased from Sigma Chemical Co.

### 2.2. Culture of the BJ Cell Line

The BJ cell line (ATCC number CRL-2522) was kindly supplied by Paula Marques and João Malva (Life Sciences Department and Center for Neurosciences and Cell Biology, Coimbra University, Portugal). This cell line was established from skin taken from normal newborn human foreskin fibroblasts. The BJ cell line has the capacity to proliferate to a maximum of 72 population doublings before the onset of senescence.

BJ cells were cultured in endotoxin-free Dulbecco's modified eagle medium (DMEM) supplemented with 10% (v/v) of inactivated fetal calf serum, 3.02 g/l sodium bicarbonate, 100 U/mL penicillin, 100 *μ*g/mL streptomycin, and 30 mM of glucose, in a humidified incubator with 5% CO_2_/95% air, at 37°C. BJ fibroblasts have a doubling time of about 72 h and were used after reaching 80–90% confluence, which occurred approximately every 7 days. Along the experiments, cells were monitored by microscopic observation in order to detect any morphological change.

### 2.3. Western Blot

The cells (a confluent 75 cm^2^ flask) were washed with ice-cold PBS and harvested in a sonication buffer. Cell lysates and protein quantification were performed as previously described [12]. NTR1, NTR2, and NTR3 levels were evaluated by Western blots. Proteins were separated by electrophoresis and transferred to PVDF membrane, as previously described [12]. The immune complexes were detected by membrane exposure to the ECF reagent, followed by scanning for blue excited fluorescence on the VersaDoc (Bio-Rad Laboratories, Amadora, Portugal). Membranes were stripped and reprobed with the antibody for *β*-actin. The generated signals were analysed using the Image-Quant TL software.

### 2.4. RNA Extraction

Cells (8 × 10^5^) were plated in 60 mm dishes in a final volume of 6 mL and were treated with 10 nM of NT during 30 h, or pretreated with 10 nM of NT during 24 h, and then stimulated with 1 *μ*g/mL of LPS during 6 h, treated for 6 h with LPS, or left untreated (control). Total RNA was isolated from these cells with TRIzol according to the manufacturer's instructions. The RNA concentration was determined by OD260 measurement using a Nanodrop spectrophotometer (Wilmington, DE, USA). RNA was stored in RNA Storage Solution (Ambion, Foster City, CA, USA) at −80°C.

### 2.5. Real-Time RT-PCR

One microgram of total RNA was reverse-transcribed using High Capacity cDNA Reverse Transcription (RT), from Applied Biosystems. Real-time RT-PCR was performed in a 20 *μ*L volume containing 2.5 *μ*L cDNA (25 ng), 10 *μ*L 2X SYBR Green Supermix, 2 *μ*L of each primer (250 nM), and 1 *μ*L of H_2_O PCR grade. Samples were denatured at 95°C for 3 min. Subsequently, 40 cycles were run for 10 sec at 95°C for denaturation, 30 sec at the appropriate annealing temperature, and 30 sec at 72°C for elongation. Real-time RT-PCR reactions were run in duplicate for each sample on a Bio-Rad My Cycler iQ5. After amplification, a threshold was set for each gene and Ct-values were calculated as previously described [12].

Primers were designed using Beacon Designer Software v7.2, from Premier Biosoft International, and thoroughly tested. Primer sequences are given in Table 1. The results were normalized using a reference gene, hypoxanthine phosphoribosyltransferase 1 (HPRT-1). This reference gene was previously validated in our lab [17] and its expression did not suffer variations upon BJ cell line stimulation with LPS and NT.

### 2.6. Immunocytochemistry Assay

Cells (2 × 10^5^) were cultured in 24-well plates containing circular glass lamella on the bottom. Cells were washed with ice-cold PBS, fixed with 4% paraformaldehyde in PBS for 10 min, and then permeabilized with 0.1% Triton X-100 in PBS containing 200 mM glycine for 5 min. After a blocking step with PBS/1% BSA for 30 min, cells were incubated overnight with antibodies against NTR1, NTR2, and NTR3 (1 : 100 in PBS containing 0.1% BSA). After washing with PBS, cells were incubated with Alexa Fluor 488-conjugated goat anti-rabbit antibody (1 : 500) and Alexa Fluor 555 phalloidin antibody (1 : 500) for 30 min at room temperature. After a washing step, cells were then incubated for 1 min with DAPI (0.1 *μ*g/mL in PBS) and mounted with Vectashield medium to reduce photobleaching. For image acquisition, fluorescence labelling was visualized using a fluorescence microscope—Zeiss Axiovert 200—and images captured with a coupled AxioCam HR camera. In each experiment, the optimal acquisition parameters were defined for the control cells and maintained for all the other conditions within the same experiment.

### 2.7. Statistical Analysis

The results were statistically analysed using the nonparametric Kruskall-Wallis test followed by Dunn's posttest, using Graphpad software. Results are presented as mean ± SD and the significance level was *P < 0.05, **P < 0.01, and ***P < 0.001.

## 3. Results

### 3.1. Expression of Neurotensin and Its Receptors on Skin Fibroblasts

BJ cells do not constitutively express neurotensin, under either basal conditions or LPS stimulation (data not shown).

**Figure 1:**
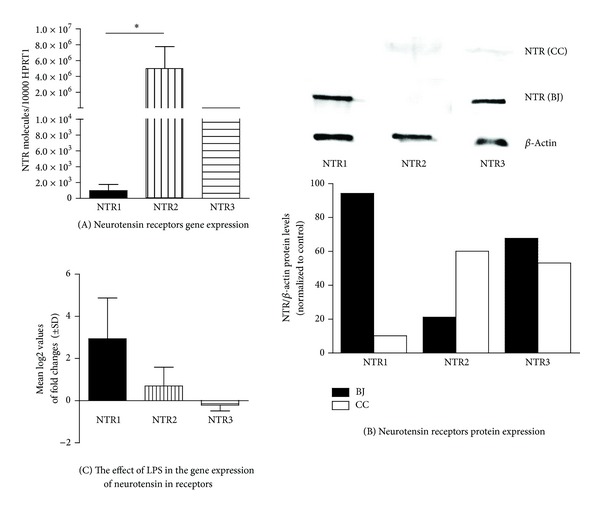
Neurotensin receptor mRNA and protein expression. Cells were maintained in medium (A, B) or treated with 1 *μ*g/mL of LPS for 6 h (C), at 37°C, with 5% CO_2_. Total RNA was isolated and retrotranscribed as indicated in Section 2. The mRNA levels were assessed by quantitative real-time RT-PCR. Gene expression is indicated as genes studied/10,000 molecules of the reference gene HPRT1 (A) or mean log2 values of fold changes relative to the control (C). Each value represents the mean ± SD from three independent experiments (*P < 0.05; Kruskall-Wallis test followed by Dunn's multiple comparison posttest). Cells extracts from untreated BJ cells (BJ) and from the murine cortical cortex (CC) were subjected to Western blot analysis (B) using NTR1, NTR2, and NTR3 antibodies, with normalization to *β*-actin. The blot shown is representative of 3 independent experiments yielding similar results.

The expression of neurotensin receptors, namely, NTR1, NTR2, and NTR3, was determined by real-time RT-PCR, Western blot, and immunocytochemistry analyses. BJ cells constitutively express the genes for NTR1, NTR2, and NTR3, with NTR2 being the most abundant, as shown in Figure 1(A). However, only the NTR1 and NTR3 were effectively transduced to protein, as observed in Figure 1(B), indicating possible cellular posttranscription modification. Brain homogenates from the cortical cortex of mice were used as positive controls for NTR reactivity, since they express all neurotensin receptors, as determined by Western blot analysis (Figure 1(B)).

In addition, the expression of neurotensin receptors was studied under inflammatory conditions. The stimulation of BJ cells with LPS for 6 h caused an increase in NTR1 and NTR2 gene expression of 2.9 ± 1.9- (*P < 0.05, *n* = 3) and 0.7 ± 0.9- (*n* = 3) fold above control, respectively. However, the expression of NTR3 slightly diminished by 0.2 ± 0.3- (*n* = 3) fold, as shown in Figure 1(C).

**Figure 2:**
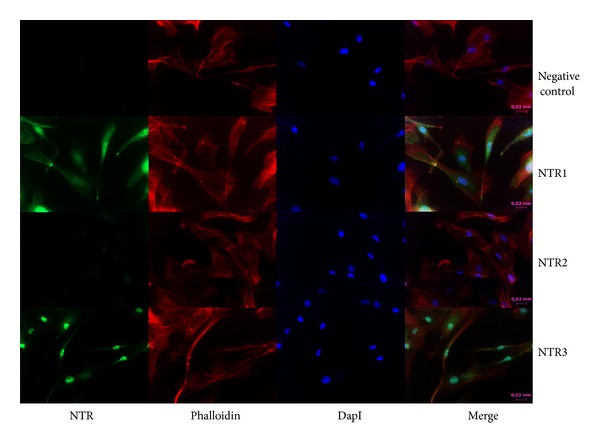
Neurotensin receptor localization. Cells were subjected to immunocytochemistry analysis as described in Section 2 using NTR1, NTR2, and NTR3 antibodies.

Neurotensin receptor localization was measured in these cells and it was verified that NTR1 is localized at the cell membrane, in the cytoplasm and in the nucleus, while NTR3 is mainly found in the nucleus (Figure 2).

### 3.2. Modulation of Gene Expression in Fibroblasts by LPS

To evaluate the expression of different genes under homeostatic conditions and upon 6 h of LPS exposure, real-time RT-PCR was performed for CCL4, CCL5, CCL11, IL-8, IL-1*β*, IL-6, and epidermal growth factor (EGF).

**Figure 3:**
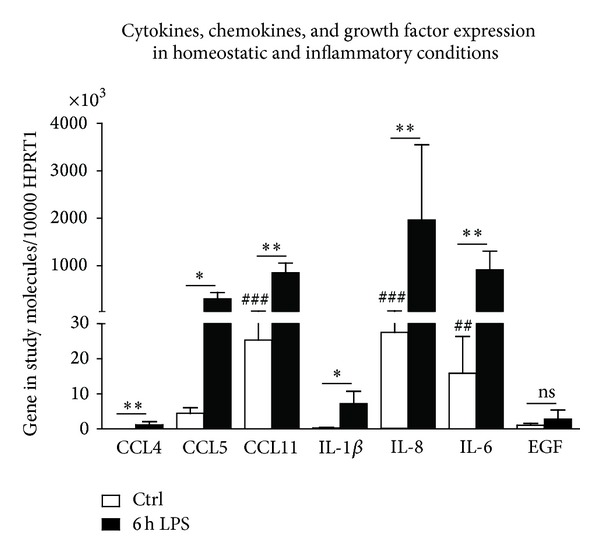
Expression of cytokines, chemokines, and growth factors in BJ cells, under homeostatic and inflammatory conditions. Cells were plated at 8 × 10^5^ cells/dish in 60 mm dishes in a final volume of 6 mL of medium and treated with 1 *μ*g of LPS during 6 h (LPS), or left untreated (Ctrl), at 37°C, with 5% CO_2_. Total RNA was isolated and retrotranscribed as indicated in Section 2. The mRNA levels were assessed by quantitative real-time RT-PCR. Gene expression is indicated as genes studied/10 000 molecules of the reference gene HPRT1. Values represent the mean ± SD from four independent experiments. Kruskall-Wallis test followed by Dunn's posttest statistical analysis was performed between cytokines, chemokines, and growth factor expression under homeostatic and inflammatory conditions (**P < 0.01; ***P < 0.001) and among cytokines, chemokines, and growth factor expression under homeostatic conditions (^##^ < 0.01; ^###^ < 0.001). IL: interleukin; EGF: epidermal growth factor.

Nonstimulated BJ cells constitutively expressed all chemokines, cytokines, and the growth factor EGF, with IL-6, IL-8, and CCL11 being the most expressed genes, by 1.6 × 10^4^ ± 1.1 × 10^4^ (^##^ < 0.01,  *n* = 4), 2.8 × 10^4^ ± 1.9 × 10^4^ (^###^ < 0.001, *n* = 4), and 2.5 × 10^4^ ± 1.7 × 10^4^ (^###^ < 0.01, *n* = 4), respectively, relative to HPRT1 gene expression, while CCL4 was barely expressed, by 6.4 ± 5.0 (*n* = 4), relative to HPRT1 gene expression (Figure 3). When stimulated with LPS, the expression levels of all these genes were highly upregulated, including the expression of chemokine CCL5 (*P < 0.05), IL-1*β* (*P < 0.05, *n* = 4), CCL4 (**P < 0.01, *n* = 4), CCL11 (**P < 0.01, *n* = 4), IL-8 (**P < 0.01, *n* = 4), and IL-6 (**P < 0.01, *n* = 4), relative to nonstimulated cells (Figure 3), reinforcing the role of fibroblasts as skin immunomodulators.

### 3.3. Modulation of Gene Expression in Fibroblast by Neurotensin

**Figure 4:**
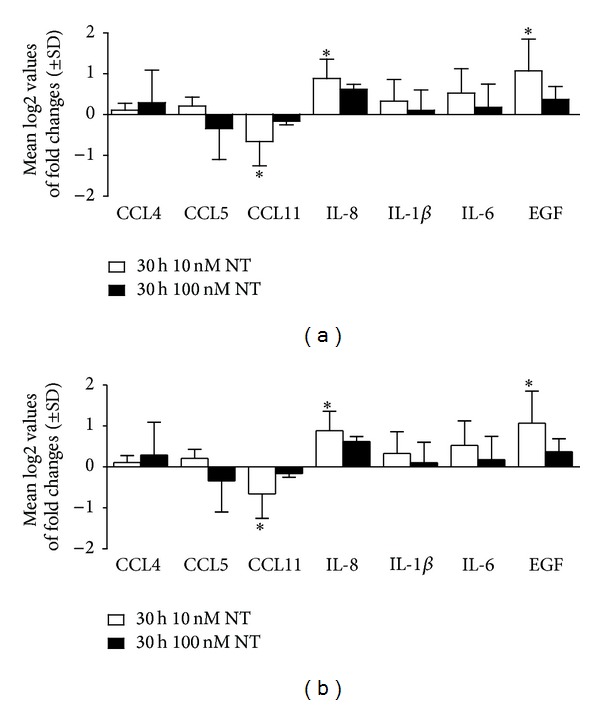
Modulation of gene expression by neurotensin under homeostatic and inflammatory conditions in BJ cells. Cells were maintained in culture medium (Ctrl) and treated with 10 nM of NT (white bars) or 100 nM of NT (black bars) during 30 h (a) or pretreated with 10 nM (white bars) and 100 nM (black bars) of NT during 24 h and stimulated with 1 *μ*g of LPS during 6 h (b), at 37°C, with 5% CO_2_. Total RNA was isolated and retrotranscribed as indicated in Section 2. The mRNA levels were assessed by quantitative real-time RT-PCR. Gene expression is indicated as mean log2 values of fold changes relative to the control. Each value represents the mean ± SD from four independent experiments (*P < 0.05; Kruskall-Wallis test followed by Dunn's posttest statistical analysis between 10 and 100 nM of NT, 10, and 100 nM of NT with the corresponding control, under homeostatic and inflammatory conditions).

To evaluate the effect of neurotensin on key inflammatory cytokines and chemokines, BJ cells were incubated with the neuropeptide alone, or with the inflammatory stimulus of LPS alone, or were incubated with the combination of both neurotensin and LPS (Figure 4). Indeed, cells were treated with (1) either 10 or 100 nM of NT for 30 h; (2) incubated with LPS alone for 6 h; (3) incubated with either 10 or 100 nM of NT for 24 h before an additional stimulus of LPS for 6 h; or (4) left untreated (control). To understand the role of neurotensin in the skin, specifically in skin fibroblasts, cells were subjected to different neurotensin concentrations (10 and 100 nM) in and out of an inflammatory environment (LPS stimulus). In fact, to better comprehend the role of neurotensin in an inflammatory environment, cells were firstly pretreated with NT and subsequently stimulated with LPS. Furthermore, this protocol may simulate an* in vivo* NT treatment immediately after an injury (before inflammation starts). After these incubation periods, total RNA was isolated, quantified, and reverse-transcribed to cDNA to finally perform real-time RT-PCR.

BJ cells treated with NT (10 nM) significantly increased the expression of EGF and IL-8 by 1.07 ± 0.45- (*P < 0.05, *n* = 3) and 0.88 ± 0.48- (*P < 0.01, *n* = 3) fold above control, respectively, while decreased CCL4 expression by −0.59 ± 0.27- (*P < 0.01, *n* = 3) fold below the control (Figure 4(a)). However, neither the cytokine nor the chemokine profile of these cells showed significant differences in expression when cells were incubated with 100 nM of NT (Figure 4(a)). However, when cells were pretreated with NT (10 nM) during 24 h followed by a 6 h LPS stimulus, both the cytokine and chemokine profiles of these cells were significantly modulated. When cells were stimulated with both 10 nM of NT and LPS, the expression of CCL4 was significantly decreased by 0.59 ± 0.28- (*P < 0.05,   *n* = 3) fold, relative to the control, as shown in Figure 4(b). In addition, when cells were incubated with 100 nM of NT plus LPS, the expression of cytokines IL-6 was significantly reduced by 1.09 ± 0.87 (*P < 0.05, *n* = 4), relative to cells treated with NT (10 nM). In contrast, the chemokine CCL11 showed a significant increase in expression of 0.65 ± 0.43- (*P < 0.05,  *n* = 3) fold relative to cells treated with NT (10 nM). Furthermore, EGF expression was significantly increased by 0.99 ± 0.785- (*P < 0.05,  *n* = 3) fold relative to the control (Figure 4(b)). In conclusion, we observed that, in the presence of neurotensin (10 nM), BJ cells presented a decrease in CCL11 chemokine expression and an increase in IL-8 cytokine expression. Under inflammatory conditions and in the presence of neurotensin, BJ cells presented a decrease in CCL4 and IL-6, while an increase in CCL-11 expression. Furthermore EGF expression was increased in cells incubated with NT either alone or in combination with LPS.

## 4. Discussion

BJ cells expressed the NTR1 and NTR3; NTR1 was located at the cell membrane, cytoplasm, and nucleus while NTR3 was exclusively located in the nucleus. As NTR3 is exclusively localized in the nucleus, its mediated responses can only occur by intracellular neurotensin. Regarding that neurotensin is not expressed by BJ cells, as they do not express NT, NT-NTR3 signalling pathway can only be activated by endocytosed neurotensin previously produced by neighbouring skin cells, such as keratinocytes. Furthermore, NT-NTR3 mediated response will have a later effect in comparison to NT-NTR1 mediated response because NTR1 is located at the cell membrane and the activation of this signalling pathway does not require NT internalization. Furthermore, upon NT binding to its receptors, NT activates different signalling pathways and cell responses, such as chemokine and cytokine expression, as previously described by us for a dendritic cell line and by others [4, 9, 18–25].

Neuropeptides receptor expression under an inflammatory environment has been shown to be regulated by neuropeptides, as observed in rat macrophages for substance P receptors [26] and in HUVECs for NPY receptors [27]. Indeed, we determined if the expression of neurotensin receptors differed in an inflammatory environment and, after cell exposure to LPS, the gene expression of NTR1 was induced while the expression of NTR3 decreased. These results suggest that the upregulation of neurotensin receptors in an inflammatory environment (LPS) will lead to a rise on NT-mediated effects and propagation of NT signalling pathways. Although endogenous NT was not expressed in BJ cells, exogenous NT produced by neighbouring cells can play an important role in NTR activation.

Regarding NT effects on the immunomodulatory function of BJ cells, NT downregulated the immunomodulatory ability of BJ cells under inflammatory conditions and significantly upregulated EGF expression, a crucial growth factor involved in cell growth and wound healing [28, 29].

Under homeostatic conditions, exogenous NT was able to significantly upregulate IL-8 and downregulate CCL11 in these cells. These cytokines are involved in the important process of immune cell recruitment to the site of inflammation [30]. IL-8 is a neutrophil-activating cytokine which induces chemotaxis and the release of granule enzymes [31] while CCL11 (or eotaxin) is an important eosinophil chemoattractant which also recruits basophils, Th2 lymphocytes, and tryptase-chymase mast cells [32–35]. Since fibroblasts orchestrate and respond to inflammatory cascades, NT upregulation of IL-8 could activate neutrophils while downregulation of CCL11 chemokine expression could decrease leukocyte recruitment in homeostatic conditions.

Under an inflammatory environment (LPS treatment), NT seemed to have the opposite effect in respect to chemokine/cytokine profile, significantly downregulating the chemoattractant CCL4 and proinflammatory cytokine IL-6. Meanwhile, NT also upregulated CCL11. Besides being a chemoattractant, CCL11 may also induce eosinophil degranulation [36] and IgE-independent degranulation of basophils [37], promoting adaptive immune responses, through the selective recruitment of Th2 lymphocytes, which is dependent on the expression of CCR4 and CCR8 on Th2 cells [38]. Indeed, the increased expression of CCL11 mediated by NT in fibroblasts could be involved in a Th2 polarized response.

Considering epidermal growth factor, a cell growth and wound healing growth factor, its expression was significantly enhanced, which is in accordance with our previous study performed in dendritic cells [12]. In fact, this same effect was already observed in granulation fibroblasts by the neuropeptide substance P [39]. EGF increase may potentially be triggering autocrine effects on BJ cells as well as paracrine effects on keratinocytes, by modulating epidermal proliferation and differentiation [40], also emphasising the importance of the EGF effect in the wound closure of wound healing [28, 29].

## 5. Conclusion

These results report the effect of exogenous NT under both homeostatic (without LPS) and inflammatory conditions (with LPS) in BJ cells. Under inflammatory conditions, NT was able to downregulate the chemoattractant function of fibroblasts, vital for the last phases of wound healing, which include its migration-proliferation and remodelling phases. Downregulation of chemokines triggered by exogenous NT could decrease the inflammatory status of the wound and could beneficially promote migration of fibroblasts to the wound site, with consequent expression of ECM proteins, important for skin repair. Furthermore, EGF-mediated effects on fibroblasts and keratinocytes will be fulcra for the remodelling phase of wound healing.

Indeed, delayed wounds like diabetic wound healing, characterized by a proinflammatory cytokine profile [1], could potentially be treated with NT, promoting the transition of the inflammatory phase to the last phases of wound healing, thus improving wound healing. Overall our results suggest that neurotensin may be of great value in therapeutic approaches for inflammatory skin diseases, through promoting wound healing. However, our* in vitro* model has limitations and treatment of fibroblasts* in vitro* with NT may not mimic its biological processes in their native environment, not taking into account the neuroendocrine, for example, sympathetic and parasympathetic, control of tissues. Thus, these findings should be confirmed with* in vivo* experiments. Meanwhile, we can hypothesize that* in vivo* NT administration, under similar conditions, may promote a decreased inflammatory response through these cells, which may be important in the later phases of healing.* In vivo* studies are needed to further confirm the potential application of NT as a therapy for diabetic foot ulcers.

## Figures and Tables

**Table 1:** Primer sequences for targeted cDNAs.

Primer	5′-3′ sequence (F: forward; R: reverse)	RefSeqID
HPRT1	F: TGACACTGGCAAAACAATG R: GGCTTATATCCAACACTTCG	NM_000194
NT	F: GCATACATCAAAGATTAGT R: TAAAGCAGTAGGAAGTTT	NM_006183
NTR1	F: GTCGTCATACAGGTCAAC R: GATGATGGTGTTCAGGAC	NM_002531
NTR2	F: GCAAGAATGAACAGAACA R: GAATGATTAGTGATGAGGTT	NM_012344
NTR3	F: TGGGTTGGAGATAGCACTGG R: ACGACTTCCTCCAGACACCT	NM_002959
IL-1*β*	F: GCTTGGTGATGTCTGGTC R: GCTGTAGAGTGGGCTTATC	NM_000576
IL-6	F: TCTGGATTCAATGAGGAGACTTG R: TCACTACTCTCAAATCTGTTCTGG	NM_000600
IL-8	F: CTTTCAGAGACAGCAGAG R: CTAAGTTCTTTAGCACTCC	NM_000584
CCL11	F: ACCAGAGCCTGAGTGTTG R: TGCCCTTTGGACTGATAATGA	NM_002986
CCL4	F: CGCCTGCTGCTTTTCTTACAC R: CAGACTTGCTTGCTTCTTTTGG	NM_002984
CCL5	F: CAGTGAGCTGAGATTGTG R: TTTGTTGTTGTTGTTGTGA	NM_002985
EGF	F: TCAGAAGATAACATTACAGAAT R: AATACACCGAGCATACAT	NM_001178130

## Abbreviations

NT: Neurotensin
IL: Interleukin
EGF: Epidermal growth factor
ECM: Extracellular matrix
WH: Wound healing
LPS: Lipopolysaccharide
CNS: Central nervous system
NTR: Neurotensin receptor
GPCRs: G protein coupled receptors
TNF: Tumor necrosis factor
BCA: Bicinchoninic acid
PVDF: Polyvinylidene difluoride
ECF: Enhanced chemifluorescence
DEPC: Diethyl pyrocarbonate
DMEM: Dulbecco's Modified Eagle Medium
RT-PCR: Reverse transcription polymerase chain reaction
HPRT-1: Hypoxanthine phosphoribosyltransferase 1.
